# Analysis of the Light Transmission Ability of Reinforcing Glass Fibers Used in Polymer Composites

**DOI:** 10.3390/ma10060637

**Published:** 2017-06-10

**Authors:** Gergely Hegedűs, Tamás Sarkadi, Tibor Czigány

**Affiliations:** 1 Department of Polymer Engineering, Faculty of Mechanical Engineering, Budapest University of Technology and Economics, Műegyetem rkp. 3, H-1111 Budapest, Hungary; hegedus@pt.bme.hu; 2 Department of Atomic Physics, Faculty of Natural Sciences, Budapest University of Technology and Economics, Budafoki út. 8, H-1111 Budapest, Hungary; sarkadi@eik.bme.hu; 3 MTA–BME Research Group for Composite Science and Technology, Műegyetem rkp. 3, H-1111 Budapest, Hungary

**Keywords:** reinforcing glass fiber, light transmission, sensor, structural health monitoring, attenuation coefficient, polymer resins, refractive index

## Abstract

This goal of our research was to show that E-glass fiber bundles used for reinforcing composites can be enabled to transmit light in a common resin without any special preparation (without removing the sizing). The power of the transmitted light was measured and the attenuation coefficient, which characterizes the fiber bundle, was determined. Although the attenuation coefficient depends on temperature and the wavelength of the light, it is independent of the power of incident light, the quality of coupling, and the length of the specimen. The refractive index of commercially available transparent resins was measured and it was proved that a resin with a refractive index lower than that of the fiber can be used to make a composite whose fibers are capable of transmitting light. The effects of temperature, compression of the fibers, and the shape of fiber ends on the power of transmitted light were examined. The measurement of emitted light can provide information about the health of the fibers. This can be the basis of a simple health monitoring system in the case of general-purpose composite structures.

## 1. Introduction

Composites are becoming more and more popular [1,2], and this necessitates the development of new testing methods, preferably non-destructive in situ methods. Most non-destructive evaluation methods require bulky, non-movable equipment, which cannot test the composite during regular operation. There are, however, methods which involve sensors built into the composite that evaluate the signals they produce to detect deformation or structural damage. These methods can be used to test the structure during normal operation and provide a way to continuously monitor the health of the structure, without the need to remove the part for testing. The sensors can be classified according to the principle of operation: electrostatic sensors, piezoresistive sensors, piezoelectric sensors [3], optical sensors, etc. [4]. A property of the light (intensity, phase, polarization, wavelength, timing, spectral distribution) travelling in the optical fiber of the optical sensor changes as a result of external influences, such as a load on the fiber, deformation, or temperature. The optical sensors used in composites can be classified based on several aspects [5,6]. Compared to electric sensors, they have several advantages: they are not disturbed by electromagnetic waves (e.g., radio waves, background radiation), therefore they do not need electromagnetic shielding, and optical fibers does not produce electromagnetic interference, either [7,8]. Their material is highly corrosion-resistant and can withstand high temperatures, therefore the sensor can be used for monitoring the manufacturing process [9,10] and structural health monitoring of composites [11] or even civil structures [12]. The sensors can be easily installed due to their small size, small mass, and flexibility. The considerably larger signal-processing equipment need not be built into the structure, therefore it does not affect the integrity of the structure. The disadvantages are that when sensors are broken, they are difficult to repair due to their small size, and the continuous structure and thus the mechanical properties of the composite change as the thickness of the built-in optical fibers is one order of magnitude higher than that of the reinforcing fibers [13]. Light travels in optical fibers due to total internal reflection. An optical fiber usually consists of a core with a refraction index n_2_, and a cladding which has a lower refraction index n_1_. Light entering the fiber reaches the boundary at an angle higher than the critical angle and is therefore reflected. Light leaves at the other end of the fiber [14].

The reinforcing fibers of composites differ considerably from optical fibers due to their different composition and manufacturing technology, but they can also be prepared to transmit light. If the reinforcing fiber is surrounded by a material with a lower refractive index, total internal reflection can occur and the fiber can transmit light. Glass reinforcing fibers can be used to transmit light and a change in the intensity of the light can give information about the health of the bundle, and therefore the composite structure (e.g., in the case of fiber fracture) [15,16]. Such a method makes in situ health monitoring possible without a noticeable change in the mechanical properties of the composite structure. If the reinforcing fibers of the composite themselves are used, sensors need not be built in, which also reduces cost.

Hayes et al. [16] successfully detected impact damage by transmitting light through unsized, commercially available Quartzel fibers coated with a resin of low refractive index. Several authors [15,17,18,19,20] examined the light transmission properties of unsized E-glass reinforcing bundles in various special-use resin systems of low refractive index, since E-glass fibers are cheaper than quartz fibers. They showed that the intensity of the transmitted light changed as a result of loading. Malik et al. [19,21] investigated the light transmission properties of custom-made small-diameter optical fibers built into composites during tensile tests. The size of the optical fibers was similar to the size of the reinforcing fibers. The advantage of optical fibers over E-glass fibers is that they can transmit light over far greater distances than a meter; therefore, a light source of lower power can be used. Their disadvantage is that they require special preparation. 

In the papers published so far, the light transmitting properties of unsized reinforcing bundles and custom-made optical fibers have been investigated in special-use resins. It has been proved that they are suitable for monitoring the health of composite structures, when the sizing is removed. It should be noted, however, that the sizing ensures a protection against the further mechanical processing steps (i.e., weaving); it has a provisional protective function, it holds the fibers together, and it provides good connection between the fiber and matrix materials.

This article presents the light transmitting ability of E-glass reinforcing bundles, and shows that this ability can be used in a common resin matrix, without special preparation (removing the sizing). Our goal is to determine the attenuation coefficient of a reinforcing bundle in a given matrix, at a given temperature and given wavelength.

## 2. Experimental Methods 

### 2.1. Reinforcing Glass Fibers

The conventional reinforcing fiber used in this study was a continuous boron-free E-CR glass bundle (Advantex T30 R25H-1200 TEX, Owen’s Corning, Watermael-Boitsfort, Belgium). The refractive index of the glass was 1.56–1.57, and the density was 2.62 g/cm^3^ according to the datasheet. The average diameter of the fibers was 15 μm.

### 2.2. Matrix Materials

Reinforcing fibers can act as light guides, if the refractive index of the surrounding materials is lower than the refractive index of the fibers. Datasheets rarely include the refractive index of resins therefore it was measured with an Abbe refractometer (AR4, A.Krüss Optronic, Hamburg, Germany). Smooth (polished on one side), 8 mm × 10 mm × 20 mm specimens were made from different transparent resins. We chose nearly water-clear transparent resins to monitor the macro-level phenomena from the outside with the naked eye. Table 1 shows the measured refractive index of the resins.

The refractive index of the MR3012 epoxy resin (Ipox chemicals, Laupheim, Germany) and MH3122 hardener (Ipox chemicals) at a weight ratio of 100:40 and 100:25 was over 2% less than the refractive index of the reinforcing glass fibers. The mechanical properties of this resin system can be varied in a wide range depending on the mixing ratio. In the experiments, this epoxy-resin system was used in these two mixing ratios.

### 2.3. Equipment Used and Measurement Layout

**Figure 1:**
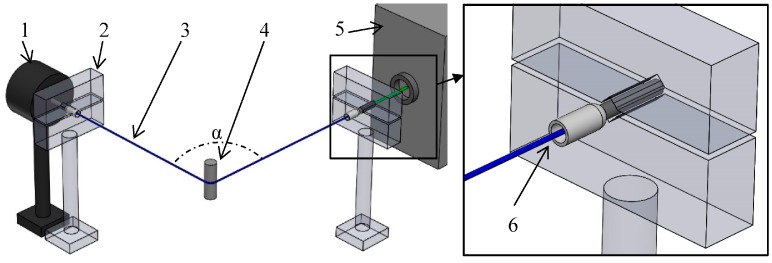
Measurement layout (1—light power meter, 2—clamp, 3—reinforcing fiber bundle, 4—bending, 5—light source, 6—cord-end terminal).

The basic layout of the experiments is presented in Figure 1.

The reinforcing fiber bundle (3) was investigated in a shrink-on tube or in the resin. The back and front segment of the bundle was fastened together with a shrink-on tube shrunk to an inner diameter of 0.8 mm over a length of 15 mm. The bundle was cut with a blade, perpendicular to the axis of the fibers to enable light to enter. With an insulated cord-end terminal (6), the bundle was secured in a clamp (2) on both sides. The bundle was bent 90° (α) so that the light from the light source can only reach the measuring equipment through the glass fibers, as direct light from the light source does not reach the measuring equipment, and thus does not influence the measured values. Two kinds of light source (5) were used: LED (XP-C LEDs, XLamp, Cree, Durham, NC, USA) and green Nd:YAG laser (532 nm wave length, dpgc-2250, Shanghai Uniwave Technology Co. LTD. (Suwtech), Shanghai, China). The emitted light was examined with a USB microscope camera (Bresser, Germany), and with a high sensitivity optical power sensor (1) (OP-2VIS, Coherent, Santa Clara, CA, USA).

It is necessary to choose an ideal wavelength light source. Kister et al. [1] showed in their work that in the visible spectrum (between 400 and 900 nm), the minimum absorption was around 500 nm for the reinforcing E-glass fibers they investigated. The maximum length of glass fibers that emitted measurable optical power was ~0.5 m. With an easy and fast method, we came to the conclusion that 532 nm wavelength green light was optimal in our case, too. A 200 mm long reinforcing fiber bundle was illuminated with blue (460 nm), green (530 nm), and red (625 nm) LED light. At the other end of the bundle, the emitted light was monitored with a USB microscope camera with 80× enlargement. According to the results of Kister et al., the intensity of the images in the case of various illumination colors were different, and the brightest image was obtained with green light. In the following experiments we used green (532 nm) Nd: YAG laser. This difference in transmission ability requires further investigation.

### 2.4. Specimen Performance

There are several phenomena that can affect the measurement of transmitted light, for example the compression of the fibers and the connection of the light source. 

#### 2.4.1. Compressing the Fibers of the Reinforcing Glass Fiber Bundle

**Figure 2:**
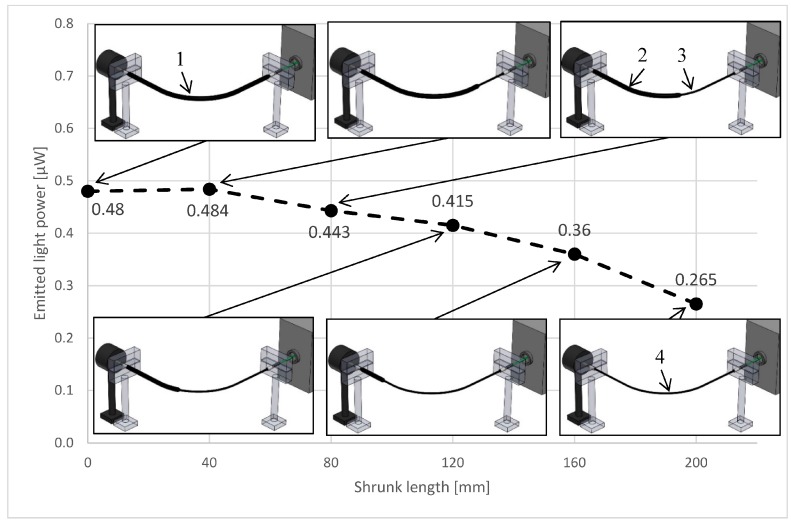
The effect of compression on emitted light power (1—reinforcing fiber bundle in the unshrunk shrink-on tube, 2—unshrunk shrink-on tube section, 3—shrunk shrink-on tube section, 4—shrunk shrink-on tube).

The rate of compression of the fibers can affect the intensity of the emitted light. The more fibers touch each other, the more light can go from one fiber into another where two fibers touch. To measure the effect of the rate of compression, a 200 mm long fiber bundle was put in a shrink-on tube. The initial inside diameter of the shrink-on tube was 1.6 mm, and the shrunk diameter was 0.8 mm. The fibers were illuminated with a green laser, and at the other end a high-sensitivity optical power sensor measured the intensity of the emitted light (similarly to 2.2). The 0.8 mm shrink-on tube compressed the fibers and could be pushed over the fibers by hand with moderate force. The 200 mm long specimen was divided into five equal segments (40 mm), and the segments were shrunk one after another. The intensity of the measured light is shown in Figure 2.

The intensity of the emitted light increased when the shrink-on tube was heated, but after the specimen got cold, the measured intensity decreased as more and more segments of the bundle were compressed by the shrink-on tube. This phenomenon can be explained with the increasing surface of the joining fibers. Where the fibers touch each other, the light can go from one fiber to the other, and this can cause loss. The experiment shows that the fibers should be compressed into a definite geometry so that the position of the fibers does not change relative to each other during the measuring process.

The measured optical power was ~10% greater when the shrink-on tube (and the fibers) were heated. It means that the transmitted optical power of the fibers is affected by the temperature. This can be utilized to monitor the temperature, or the in situ curing process during the manufacturing of the composite part [22,23]. Further investigation is needed to find out the effect of temperature on the transmitted optical power. In this work, all experiments were conducted at room temperature (22 °C).

#### 2.4.2. Connecting the Light Source

We found that the transmitted optical power greatly depends on the position of the end of the fibers, the light source, and the optical power sensor. The quality of the end of the fibers influences the amount of light entering the fibers, and so the optical power emitted at the other end (±40%). The best solution would be, just as in the preparation of optical fibers used in the telecommunication industry, to cleave the fibers perpendicular to the axis. This is not possible one by one in a bundle consisting of thousands of fibers. A modified optical connector that embeds the fibers in a resin and fine-polishes the end of the fibers could provide the best results in connecting the light source.

## 3. Results and Discussion

### 3.1. Analysis of the Light-Transmitting Ability of Glass-Fiber Bundles

**Figure 3:**
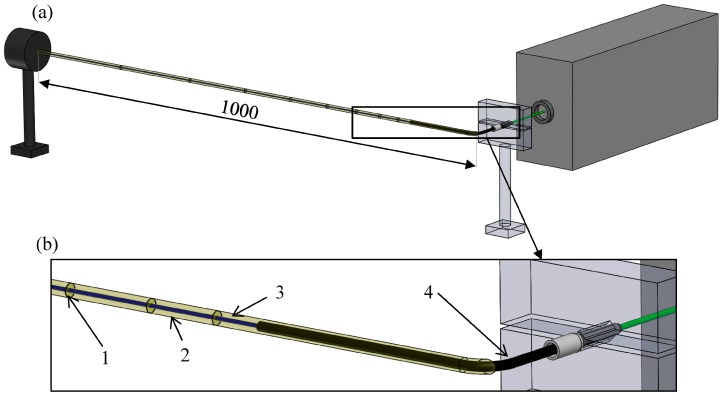
(**a**) Layout for measuring light transmission and (**b**) the specimen (1—measurement length marker, 2—resin, 3—glass fiber bundle, 4—shrink-on tube).

The aim of this investigation is to determine the dependence of the transmitted light on the length of the reinforcing glass-fiber bundle. The schematic illustration of the layout of the experiment and the size of the specimen are presented in Figure 3.

**Figure 4:**
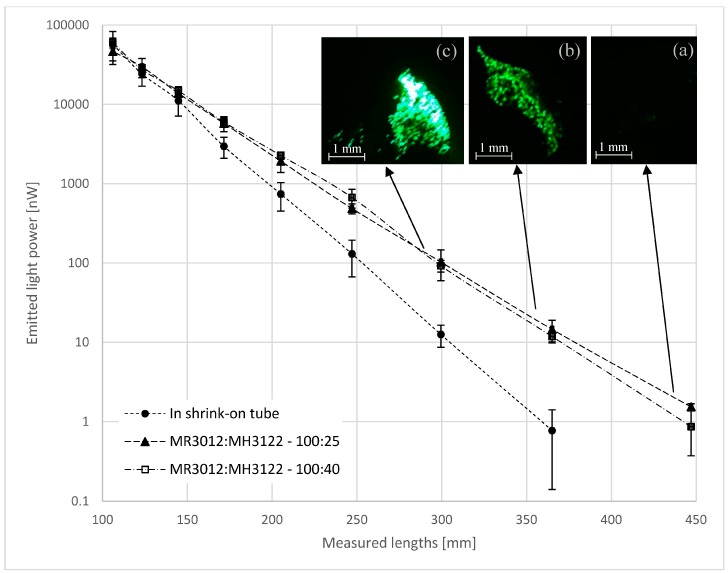
The light power emitted from the reinforcing glass fiber bundles at room temperature. The wavelength of the light was 532 nm. The bundles were in shrink-on tubes or in the MR3012:MH3122 resin (weight ratios of 100:40 or 100:25). The images of the emitted light were recorded with a USB microscope camera at a length of (**a**) 447 mm; (**b**) 365 mm; and (**c**) 300 mm.

The fiber bundle was measured without resin in the shrink-on tube. The shrink-on tube was shrunk to an inside diameter of 0.8 mm to fix the positions of the filaments. Then, the fiber bundle was built into the resin selected earlier (MR3012:MH3122; mass ratios of 100:40 and 100:25). In order to measure the transmitted light in the fibers, the specimen was bent 40 mm apart from the point of connection of the light source with a 6 mm radius. After the bend, the measurement lengths were marked on a 1-meter section. The specimen was secured in a clamp in front of the green laser light source. At the other end of the specimen, the emitted light was recorded with a USB microscope camera, and measured with a high-sensitivity optical power sensor at room temperature. The glass fiber bundle in the shrink-on tube or in the resin was cut at different lengths (starting from the end opposite the light source), and the intensity of the transmitted light was measured (Figure 4).

The intensity of the light emitted from the fiber bundle in the shrink-on tube as well in resins decreases exponentially with the distance, and in our measurement conditions we were able to detect light up to 450 mm from the light source. Kister et al. [15] achieved a comparable result by investigating the light transmission characteristics of unsized E-glass fiber bundles built into a special resin system. Our examination shows that it is not necessary to unsize the glass fibers and build them into a special optical resin to enable them to transmit light in composite materials. The transilluminated length depends on the power of the light source, the quality of the fiber ends, and the effectiveness of the light coupling. In order to exclude these effects from the characterization of the light transmission ability of reinforcing glass fibers, the emitted optical power can be measured and compared at different lengths.

### 3.2. The Attenuation Coefficients of the Reinforcing Glass Fiber Bundle

**Figure 5:**
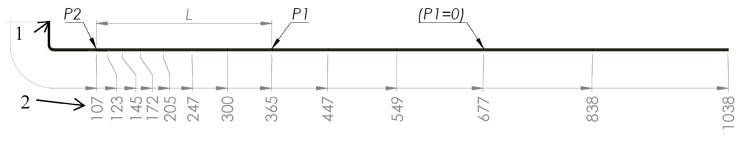
The marked and measured lengths in mm for the calculation of the attenuation coefficients (1—incident light, 2—measured lengths).

The power of light travelling through fibers decreases exponentially with the distance as a result of absorption and scattering. It can be characterized with the attenuation coefficient, which is unaffected by the power of the light source and the effectiveness of light coupling (by the amount of incident light), but depends on the temperature, the wavelength of the light source, and the materials of the fibers and resin. The attenuation coefficient (α_λ_) of the fiber bundle can be calculated from the measured values, similar to the optical fibers used in telecommunications (1) [14]:

α_λ_ = 10/L × lg(P_2_/P_1_), (1)

where P_2_ is the transmitted light power after the bent part of the specimen (it is 107 mm from the end for accessibility), P_1_ is the transmitted light power by the marked fiber sections, and L is the length between them (Figure 5). The wavelength of the light source is 532 nm (α).

**Figure 6:**
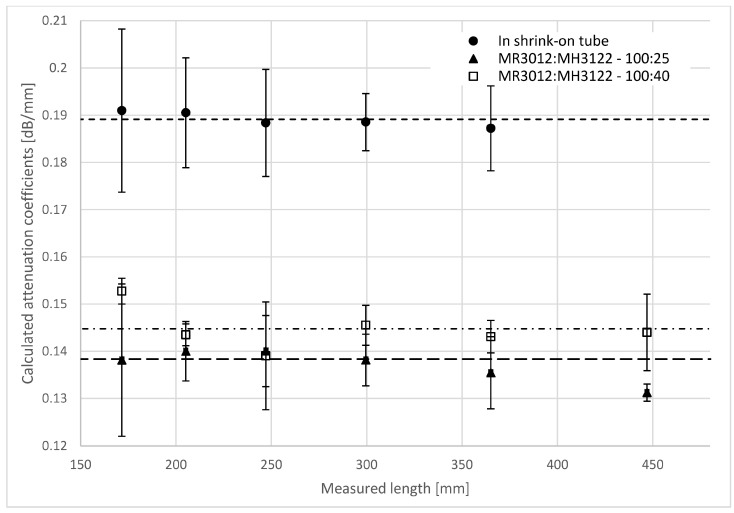
The attenuation coefficients of the reinforcing glass fiber bundles at room temperature and with a 532-nm light source.

The calculated attenuation coefficients for different lengths of the specimen are shown in Figure 6.

The attenuation coefficients of the reinforcing glass fiber bundle at room temperature and with a 532-nm light source in the shrink-on tube is 0.189 ± 0.020 dB/mm; in the MR3012:MH3122 resin at a weight ratio of 100:40 it is 0.145 ± 0.016 dB/mm; while in the MR3012:MH3122 resin at a weight ratio of 100:25 it is 0.138 ± 0.018 dB/mm. The attenuation coefficients of the bundle are lower in the resin than in the fibers compressed in the shrink-on tube. The scattering and the absorption of the transmitted light in the fiber bundle in resin is lower. The explanation for this is that there is a difference in the refractive index at the interface of the fibers and the resin, where there is total internal reflection. Thanks to this reflection, the fibers can act as light guides, similar to optical fibers. On the other hand, the compressed fibers in the shrink-on tube touch each other and the light can travel from one fiber to another, which causes far more loss. 

The attenuation coefficient can characterize the reinforcing glass fibers well in concrete circumstances (temperature, wavelength, surrounding material are given) because it is independent of the efficiency of the way light enters the bundle (the preparation of the bundle end and the position of the light source).

## 4. Conclusions

We demonstrated that the reinforcing E-CR glass fiber bundle can be adapted to transmit light in common resin without removing the sizing if the resin has a lower refractive index than the glass fibers. We measured the refractive index of commercially available transparent resins, and we measured the transmitted light power of the reinforcing fibers in a shrink-on tube, and built them into the chosen resin with a lower refractive index than that of the fibers. The intensity of the transmitted light depends on the geometry of the compressed fibers, the temperature, the position of the light source, and the preparation of the end of the bundle. The intensity of the transmitted light decreases exponentially with the length of the fiber bundle. We were able to measure optical power at up to 500 mm from the point where the light enters the specimen. The fiber bundle can be characterized with an attenuation coefficient (if the temperature, wavelength of the light source, and surrounding material are given). 

We can obtain information about the integrity of a commercially available, unprepared reinforcing bundle and thus the structural integrity of a common composite reinforced with it by monitoring the light transmitted through the fibers. The method could be used to evaluate external loads (deformation, strain, stress, tension, temperature), and therefore it could be the basis of continuous health monitoring in composite structures.

## Figures and Tables

**Table 1:** The refractive index of the examined resins.

Resin	Type	Weight Ratio	Refractive Index
epoxy	ipox^®^ ER 1010:EH2293	100:22.6	1.585
epoxy	ipox^®^ MR3008:MH3120	100:20	1.580
polyester	SYNOLITE 0328-A-1:Promox	100:2	1.567
epoxy	ipox^®^ MR 3010:MH 3124	100:33	1.565
polyester	L UP20:Promox	100:2	1.564
polyester	SYNOLITE 0564-A-1:Promox	100:2	1.553
epoxy	ipox^®^ MR3012:MH3122	100:40	1.520
epoxy	ipox^®^ MR3012:MH3122	100:25	1.505
